# Complex reconstruction after wide excision of juvenile aponeurotic fibromatoses of upper one-third of leg

**DOI:** 10.1007/s11751-014-0195-x

**Published:** 2014-07-27

**Authors:** Md Sohaib Akhtar, Rabeya Basari, A. H. Khan, Mohd Fahud Khurram

**Affiliations:** 1 Post Graduate Department of Burns, Plastic and Reconstructive Surgery, JNMC, AMU, Aligarh, UP India; 2 Department of Pathology, JNMC, AMU, Aligarh, UP India

**Keywords:** Juvenile musculoaponeurotic fibromatosis, Locally aggressive tumor, Complex reconstruction

## Abstract

Juvenile musculoaponeurotic fibromatoses are benign tumors which arise from musculoaponeurotic stromal cells. They rarely occur in lower extremity and more rarely in children. They are locally invasive tumors with a high incidence of recurrence after surgery. Hence, wide local excision is the treatment of choice for such tumors. However, complex reconstruction is often required to cover the resulting soft tissue defect. This report presents a 12-year-old boy with a juvenile musculoaponeurotic fibromatosis in the anteromedial aspect of the upper third of a left leg. Following wide local excision, two local flaps, medial gastrocnemius and a distally based peroneal artery perforator flap, were used to reconstruct the soft tissue defect. Reconstruction has provided an acceptable functional and cosmetic result.

## Introduction

Stout [1] first described the term fibromatosis that includes a wide range of non-malignant conditions that are characterized by local invasiveness and a tendency to recur after excision. Juvenile musculoaponeurotic fibromatosis is a subtype of fibromatoses. It tends to occur adjacent to skeletal muscles and has a tendency to infiltrate them. These tumors can infiltrate up to 2–3 cm beyond the palpable margins [2]. Therefore, they require wide local excision to ensure tumor-free margins. The resulting defect is usually large and requires complex reconstruction to maintain function and cosmesis. Reconstruction usually requires either free tissue transfer or complex local flap options including fasciocutaneous [3] and muscle flaps [4].

This report presents a case of juvenile musculoaponeurotic fibromatosis in a 12-year-old boy. Following a local wide excision, the defect was covered using a medial gastrocnemius flap proximally and a distally based peroneal artery perforator fasciocutaneous flap for the distal part. Since surgical margins were tumor free, no postoperative radiotherapy was required. Patient was followed for 2 years and no recurrence was observed.

## Case history

**Fig. 1:**
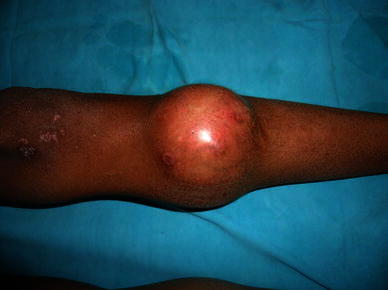
Preoperative photograph showing swelling over upper 1/3rd of left leg

A 12-year-old boy presented with an 18-month history of swelling over the proximal part of left leg (Fig. 1). Initially the swelling was small but gradually increased in size with occasional episodes of pain and increasing difficulty in walking. Physical examination revealed a firm to hard, tender swelling over the anteromedial aspect of proximal third of left leg. The swelling was fixed to overlying skin. Neurovascular structures were intact.

**Fig. 2:**
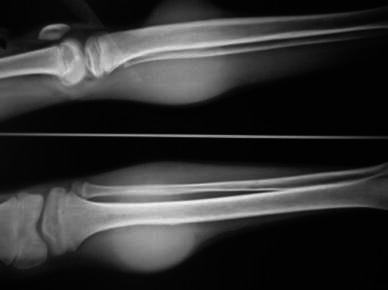
Plain radiograph showing soft tissue involvement with intact cortex

**Fig. 3:**
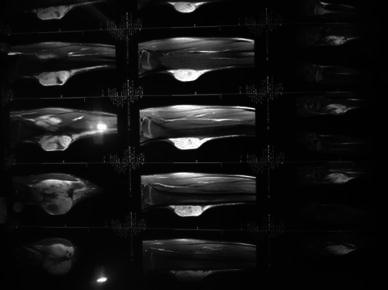
MR image showing heterogeneous, multilobulated, altered signal intensity mass lesion involving the muscular and fascial plane of the proximal left

A plain radiograph of the area revealed soft tissue expansion with no bony involvement (Fig. 2). A MRI showed a heterogeneous, multilobulated, altered signal intensity mass lesion involving the muscular and fascial plane of the proximal left leg. The tumor occupied the anteromedial aspect of the metaphyseodiaphysial region of tibia, but there was no evidence of cortical breech or intramedullary extension (Fig. 3).

**Fig. 4:**
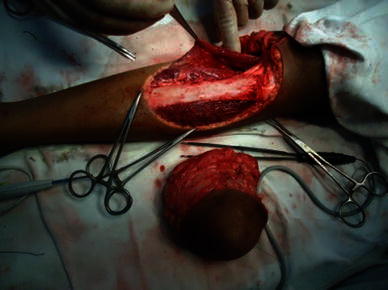
Intraoperative image after local wide excision showing large soft tissue defect of upper one-third and part of the middle one-third of leg

**Fig. 5:**
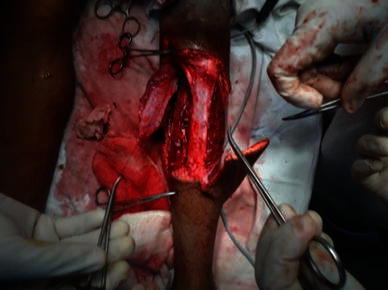
Intraoperative image showing harvest of medial gastrocnemius and inferiorly based peroneal artery perforator flap

**Fig. 6:**
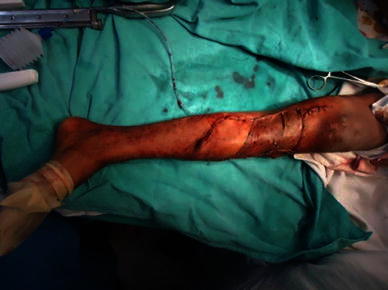
Immediate post-op photograph

The patient was operated under spinal anesthesia and tourniquet control. The tumor was excised with 3-cm margin beyond the palpable edge of the tumor (Fig. 4). The resulting defect was reconstructed using a medial gastrocnemius muscle flap for proximal cover and inferiorly based peroneal artery perforator flap with split thickness skin graft distally (Figs. 5 and 6) The leg was immobilized for 2 weeks with the knee in full extension.

**Fig. 7:**
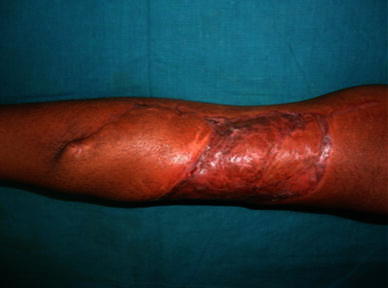
Follow-up photograph with well taken up flap and graft

Histopathological examination confirmed the diagnosis of a juvenile aponeurotic fibromatosis with negative margins and base. Hence, no post-op radiotherapy was required. No recurrence was recorded on follow-up after 2 years (Fig. 7).

## Discussion

Juvenile musculoaponeurotic fibromatoses are also termed as “aggressive fibromatoses.” These lesions encompass a wide variety of benign fibrous tissue proliferations having similar microscopic findings. The biological behavior of these tumors is intermediate between benign fibrous tissues and fibrosarcomas [5]. These tumors also referred to as desmoid tumors and represent <3 % of all soft tissue tumor with annual incidence of 0.2–0.5 per 100,000 population [6–8]. They commonly occur between the ages 15–60 years. They are more common in females [9].

CT and MRI are useful investigations to evaluate the size and extension of desmoid tumors. However, MRI is considered to be the investigation of choice for these tumors due its multiplanar imaging capabilities. It demonstrates the masses of low signal intensity relative to muscle on T1-weighted images. T2-weighted images show variable signal intensity relative to muscle [10].

Wide local excision is indicated in the management of such tumors due to their local aggressive nature. The aim of surgery is to achieve a tumor-free margin to reduce the risk of recurrence and morbidity [11]. The extent of surgical excision remains a controversial issue, and an aggressive approach to get tumor-free margins can lead to amputation of the extremity [12]. Therefore, proper planning and reconstruction is very important in such cases to avoid unnecessary morbidity and providing a functional and cosmetically acceptable result.

The presented case is a rare case of juvenile musculoaponeurotic fibromatosis in a 12-year-old boy. The case highlights the typical presentation of such tumors. The option of a free flap was excluded due to the available local options and the increased risks associated with such a major reconstruction if the more complex microvascular route was selected.

It is important that the diagnosis and tumor-free margins are confirmed intraoperatively by frozen section analysis, but this facility is unavailable in our center. The rate of local control after surgery is variable and depends on the status of the margins of resection. A control rate of 72 and 41 % has been described in the literature for tumor-negative and tumor-positive margins, respectively. Addition of postoperative radiotherapy gives better results with a local control rate of 94 and 75 %, respectively, for tumor-negative and tumor-positive margins [13]. Since the surgical margins and base were negative in the presented case, postoperative radiotherapy was not required. No local recurrence was observed after a 2-year follow-up.

## Conclusion

It is important to widely excise Juvenile musculoaponeurotic fibromatoses to get a tumor-free margin. Appropriate surgical options should be chosen for adequate coverage, to get a functional limb and to achieve an acceptable cosmetic appearance. Due to its high recurrence rate, the patient must be kept on regular postoperative monitoring.

## References

[1] Stout AP (1954). Juvenile fibromatoses. Cancer.

[2] Mackenzie DH (1972). The fibromatoses: a clinicopathological concept. Br Med J.

[3] Tolhurst DE, Haeseker B, Zeeman RJ (1983). The development of fasciocutaneus flap and its application. Plast Reconstr Surg.

[4] Mathes S, Nahai F (1982). Clinical application of muscles and musculocutaneous flaps.

[5] Chaudhuri B, Das Gupta TK, Das Gupta TK, Chaudhuri PK (1998). Pathology of soft tissue sarcomas. Tumors of the soft tissues.

[6] Reitamo JJ, Scheinin TM, Havry P (1986). The desmoid syndrome. New aspects in the cause, pathogenesis and treatment of the desmoid tumor. Am J Surg.

[7] Dahn J, Johnson N, Lundh G (1963). Desmoid tumours. A series of 33 cases. Acta Chir Scand.

[8] McAdam WAF, Goligher JC (1970). The occurrence of desmoids in patients with familial polyposis coli. Br J Surg.

[9] Hansmann A, Adolph C, Vogel T, Unger A, Moeslein G (2004). High-dose tamoxifen and sulindac as first-line treatment for desmoids tumors. Cancer.

[10] Lee J, Glazer H (1990). Controversy in the MR imaging of fibrosis. Radiology.

[11] Abbas AE, Deschamps C, Cassivi SD, Nichols FC, Allen MS, Schleck CD, Pairolero PC (2004). Chest-wall desmoid tumors: results of surgical intervention. Ann Thorac Surg.

[12] Lewis JJ, Boland PJ, Leung DH (1999). The enigma of desmoids tumors. Ann Surg.

[13] Nuyttens JJ, Rust PF, Thomas CR, Turrisi AT (2000). Surgery versus radiation therapy for patients with aggressive fibromatosis or desmoids tumors. A comparative review of 22 articles. Cancer.

